# Multi-Mode Damage and Fracture Mechanisms of Thin-Walled Tubular Parts with Cross Inner Ribs Manufactured via Flow Forming

**DOI:** 10.3390/ma17071576

**Published:** 2024-03-29

**Authors:** Xiang Zeng, Leheng Huang, Xiaoguang Fan, Hongwei Li, Mei Zhan, Zhongbao Mi, Xuefeng Xu, Yubin Fan

**Affiliations:** 1 School of Aeronautical Manufacturing Engineering, Nanchang Hangkong University, Nanchang 330063, China; zengxiang_01@126.com (X.Z.); h13907072473@126.com (L.H.);; 2 Shaanxi Key Laboratory of High-Performance Precision Forming Technology and Equipment, Northwestern Polytechnical University, Xi’an 710072, China; 3 State Key Laboratory of Solidification Processing, School of Materials Science and Engineering, Northwestern Polytechnical University, Xi’an 710072, China

**Keywords:** thin-walled tubular part, longitudinal and transverse inner ribs, flow forming, modified GTN model, damage, fracture

## Abstract

In order to study the multi-mode damage and fracture mechanisms of thin-walled tubular parts with cross inner ribs (longitudinal and transverse inner ribs, LTIRs), the Gurson–Tvergaard–Needleman (GTN) model was modified with a newly proposed stress state function. Thus, tension damage and shear damage were unified by the new stress state function, which was asymmetric with respect to stress triaxiality. Tension damage dominated the modification, which coupled with the shear damage variable, ensured the optimal prediction of fractures of thin-walled tubular parts with LTIRs by the modified GTN model. This included fractures occurring at the non-rib zone (NRZ), the longitudinal rib (LIR) and the interface between the transverse rib (TIR) and the NRZ. Among them, the stripping of material from the outer surface of the tubular part was mainly caused by the shearing of built-up material in front of the rollers under a large wall thickness reduction (Δ*T*). Shear and tension deformation were the causes of fractures occurring at the NRZ, while axial tension under a large TIR interval (*l*) mainly resulted in fractures on LIRs. Fractures at the interface between the TIR and NRZ were due to the shearing applied by rib grooves and radial tension during the formation of ribs. This study can provide guidance for the manufacturing of high-performance aluminum alloy thin-walled tubular components with complex inner ribs.

## 1. Introduction

Aluminum alloy thin-walled tubular parts with inner ribs successfully integrate a lightweight material and structure, meeting the requirements of high performance, light weight and high reliability of the aerospace field. In recent years, more and more attention has been paid to them by scholars and researchers. Due to the characteristics of high and complex ribs accompanying thin walls, the manufacture of thin-walled tubular parts with inner ribs is difficult, especially via integrated manufacturing. Thus, some new exploratory manufacturing techniques, such as the extrusion technique, have been put forward by scholars. However, the large load and thick walls formed by this technique may restrict its application in components with large size and thin-walled structures.

As an advanced plastic forming method with high forming accuracy and low forming load, flow forming holds potential as a method to achieve the rapidly integrated forming of such components. The spinning of tubular parts is commonly classified as flow forming [1]. Moreover, spinning techniques are often divided into conventional spinning and power spinning depending on the wall thickness of the spun parts. Power spinning mainly consists in the flow spinning of tubular parts and shear spinning of conical parts [2,3]. According to whether the flow direction is consistent with the rollers, spinning can be divided into forward and backward spinning. Recently, some researchers applied the flow forming technique to the forming of tubular parts with stiffened ribs. For example, Domack and Wagner [4], Abd-Eltwab [5] and Xia et al. [6] produced tubular parts with longitudinal inner ribs by a combined spin/flow forming process, ball spinning and hot backward flow forming, respectively. Luo et al. [7] used compound spinning to manufacture tubular parts with transverse/ring inner ribs. Zeng et al. [8] manufactured tubular parts with orthogonal inner ribs by flow forming.

However, the flow forming of thin-walled tubular parts with complex inner ribs is a continuous local loading process in which multiple stress states of compression, tension and shear coexist. The material undergoes repeated loading and unloading deformation, resulting in the easy occurrence of damage and fracture defects. In fact, fracture defects are also typical defects that often occur in plastic forming, especially in spinning under unreasonable parameters. For instance, Xu et al. [9] found that cracks occur at different positions of an internally toothed gear with unreasonable process parameters during multi-pass stagger spinning. Increasing the wall thinning rate and the thickness of the initial plate blank can improve the filling quality of inner teeth, but an excessive increase in wall thinning rate will lead to cracking. Within a certain range, a larger feed ratio is conducive to the filling of inner teeth and reducing the risk of fracture. Kuss et al. [10] found that the formability of tubes or rods is limited by surface cracking defects during ball spinning, but the damage can be minimized by adjusting the wall thickness reduction and axial feed rate.

Since experimental studies on spinning are time-consuming and costly, FE simulation is often employed as an alternative method. In order to reveal the fracture mechanism of spun/flow formed parts, ductile fracture models are usually embedded into FEM of spinning. Li et al. [11] accurately predicted cracking locations in shear spinning using a modified Lemaitre model in which the anisotropic effective stress of the Barlat’89 yield criterion was considered. Wang et al. [12] found that the original GTN model was suitable for predicting macroscopic cracks occurring at the material uplift area but it could not predict the damage evolution in the deformation area well due to negative stress triaxiality caused by shear deformation during forward tube spinning. Wu et al. [13] pointed out that stress triaxiality was usually less than −1/3 during tube spinning. Therefore, the original GTN model was modified considering shear damage to predict damage evolution and fracture location during tube spinning. As mentioned above, although the original GTN model can predict fractures well under high stress triaxiality, it cannot predict fractures caused by shear deformation at low stress triaxiality [14,15]. So, many scholars, such as Morin et al. [16] and Khan et. al. [17], modified the GTN to enable its application at low stress triaxiality, employing stress state characterization equations. So far, four typical stress state characterization equations have mainly been proposed by scholars to modify the GTN model, as given in Table 1. In 2008, Nahshon and Hutchinson [18] employed a normalized third deviatoric stress invariant to express the stress state characterization equation, while Xue et al. [19] defined the stress state characterization equation with a normalized Lode angle. In 2014 and 2016, Malcher et al. [20] and Jiang et al. [21] proposed two stress state characterization equations related to Lode angle and stress triaxiality, respectively. It can be seen that the former two use symmetric forms with a normalized third deviatoric stress invariant/Lode angle, while the latter two have discontinuity points or many undetermined parameters. Since the volume fraction of voids increases faster at high stress triaxiality than that at low stress triaxiality, the effect of stress triaxiality should be considered in the stress state characterization equation.

As for studies about the damage and fracture mechanisms of spinning, Wu et al. [13] found that damage evolution was related to the wall thinning rate in multi-pass tube spinning. Under a small thinning rate (<10%), tension damage led to the accumulation of total damage. With an increase in thinning rate, shear damage was greater than tension damage, which became the main cause of fractures. When the thinning rate was greater than 30%, negative stress triaxiality on the outer layer suppressed damage accumulation. Thus, the damage to the inner layer was greater than that to the outer layer, resulting in the occurrence of fractures on the inner layer of the tube. Wang et al. [22] found that the deformation in the thickness direction was uneven during backward flow forming. Plastic deformation mainly occurred in the outer layer of the tube and gradually expanded to the inner layer with increasing spinning passes. A large shear strain in the outer layer was an important cause of fracture. Similarly, Mohebbi et al. [23] found that the large equivalent plastic strain on the surface of the tube was mainly due to high shear strain, which appeared not only in the longitudinal direction, but also at the cross section during flow forming. Due to the complex structure of tubular parts with LTIRs, multi-mode damage and fractures will emerge under unreasonable processing parameters and geometric parameters of mandrel, but those have not been studied.

Thus, in this paper, the GTN model was modified via a new strategy of coupling tension damage and shear damage with a newly proposed stress state function. The modified GTN model can be suitable for multi-mode fracture prediction at low and high stress triaxiality. Then, the modified GTN model was applied in flow forming to reveal the damage and fracture mechanisms of tubular parts with LTIRs. This study will contribute to the high-performance manufacturing of aluminum alloy thin-walled tubular components with complex inner ribs.

## 2. Ductile Fracture Model

### 2.1. Stress State Characterization Equation

**Figure 1:**
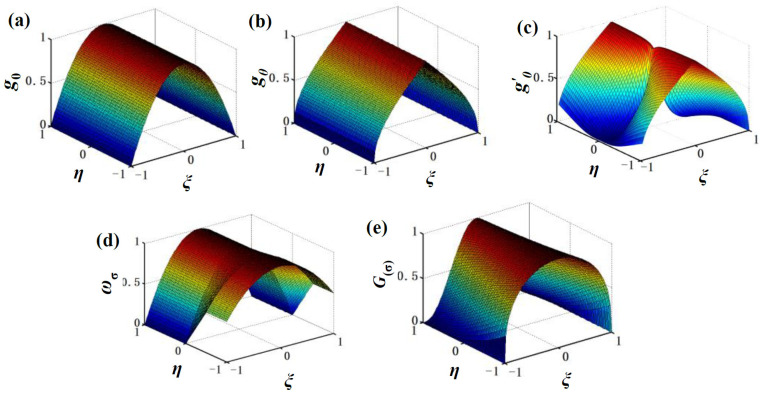
Three-dimensional diagrams of the stress state characterization function: (**a**) Nahshon’s and Hutchinson’s function [18]; (**b**) Xue’s function [19]; (**c**) Malcher’s function [20]; (**d**) Jiang’s function [21]; (**e**) new function proposed in this paper (*k*_g_ = 1).

In order to illustrate the impact of different stress triaxialities on damage, this paper proposes a new stress state characterization equation as given by Equation (1), which also incorporates Lode angle and stress triaxiality. It is in an asymmetric form with respect to stress triaxiality, as shown in Figure 1, since the growth rate of voids under high stress triaxiality is faster than that under low stress triaxiality.

(1) $$G_{\left(\sigma \right)}=(1-\xi^{2}{)}^{\exp(k_{g}\eta )}$$

where *K*_g_ is the correction parameter. ξ denotes the normalized third deviatoric stress invariant. It is given as:

(2) $$\xi ={\left(\frac{r}{\sigma_{\mathrm{eq}}}\right)}^{3}$$

(3) $$r={\left[\frac{27}{2}(\sigma_{1}-\sigma_{m})(\sigma_{2}-\sigma_{m})(\sigma_{3}-\sigma_{m})\right]}^{1/3}$$

(4) $$\sigma_{m}=\frac{1}{3}(\sigma_{1}+\sigma_{2}+\sigma_{3})$$

(5) $$\sigma_{\mathrm{eq}}=\sqrt{\frac{{\left(\sigma_{1}-\sigma_{2}\right)}^{2}+{\left(\sigma_{2}-\sigma_{3}\right)}^{2}+{\left(\sigma_{1}-\sigma_{3}\right)}^{2}}{2}}$$

where $\sigma_{\mathrm{eq}}$ and $\sigma_{m}$ are the Von Mises equivalent stress and mean stress [24], respectively. $\sigma_{1}$, $\sigma_{2}$ and $\sigma_{3}$ are the principal stresses. η is stress triaxiality [25], which is given as:

(6) $$\eta =\frac{\sigma_{m}}{\sigma_{\mathrm{eq}}}$$

### 2.2. The Evolution of Damage

Since a single damage variable cannot accurately elucidate the different damage mechanisms occurring both at high and low stress triaxiality, double damage variables are used in this paper, dividing the damage related to tension-dominant deformation (tension damage) from the damage related to shear-dominant deformation (shear damage). Namely, the change in volumetric plastic strain caused by the growth of voids in the Gurson model is reserved under high stress triaxiality, and shear damage regarded as an independent damage variable is mainly related to the deviatoric stress tensor under low stress triaxiality [20].

1. The evolution of tension damage

Tension damage (f) includes the nucleation and growth of voids caused by tension strain

(7) $$f=f_{n}+f_{g}$$

where $f_{n}$ and $f_{g}$ denote the damage induced by void nucleation and void growth under tensile loading condition, respectively.

The change in void volume fraction rate caused by tension strain [15] is given by Equation (8):

(8) $$\dot{f}={\dot{f}}_{n}+{\dot{f}}_{g}$$

where ${\dot{f}}_{n}$ and ${\dot{f}}_{g}$ denote the void nucleation rate and void growth rate under tensile loading conditions, respectively. The void growth rate is related to the volumetric plastic strain rate ${\dot{\varepsilon }}_{\nu }^{p}$:

(9) $${\dot{f}}_{g}=(1-f){\dot{\varepsilon }}_{\nu }^{p}$$

Based on the strain-controlled void nucleation rule, the void nucleation rate caused by tension strain is given by:

(10) $${\dot{f}}_{n}=A{\dot{\bar{\varepsilon }}}^{p}$$

Therein,

(11) $$A=\left\{\begin{array}{l} \frac{f_{N}}{s_{N}\sqrt{2\pi }}\exp\left[-\frac{1}{2}{\left(\frac{{\bar{\varepsilon }}^{p}-\varepsilon_{N}}{s_{N}}\right)}^{2}\right]\\ 0 \end{array}\right.\begin{array}{c} \;\eta >0\\ \;\eta \leq 0 \end{array}$$

where ${\bar{\varepsilon }}^{p}$ is the equivalent plastic strain. $f_{N}$ denotes the fraction of second phase particles with nucleation potential. $\varepsilon_{N}$ and $s_{N}$ represent the mean strain for nucleation and the corresponding standard deviation, respectively. Notably, only void nucleation at positive stress triaxiality is considered since it will not take place at negative stress triaxiality.

2. The evolution of shear damage

Shear damage (*D*) is in the same form as tension damage. Namely, shear damage includes void nucleation and growth under shearing.

(12) $$D=D_{n}+D_{\mathrm{shear}}$$

where $D_{n}$ and $D_{\mathrm{shear}}$ denote the damage induced by void nucleation and void growth under shear loading conditions, respectively.

The change rate of shear damage is shown by Equation (13):

(13) $$\dot{D}={\dot{D}}_{n}+{\dot{D}}_{\mathrm{shear}}$$

where ${\dot{D}}_{n}$ and ${\dot{D}}_{\mathrm{shear}}$ denote the void nucleation rate and void growth rate under shear loading conditions, respectively.

The change in void growth rate caused by shear strain [19] is:

(14) $${\dot{D}}_{shear}=\frac{3}{2}{\left(\frac{6}{\pi }\right)}^{1/3}D^{1/3}{\bar{\varepsilon }}^{p}{\dot{\bar{\varepsilon }}}^{p}$$

where ${\dot{\bar{\varepsilon }}}^{p}$ is the equivalent plastic strain rate. The void nucleation rate caused by shear strain is:

(15) $${\dot{D}}_{n}=A^{\prime }{\dot{\bar{\varepsilon }}}^{p}$$

Therein,

(16) $$A^{\prime }=\frac{D_{N}}{{s^{\prime }}_{N}\sqrt{2\pi }}\exp\left[-\frac{1}{2}{\left(\frac{{\bar{\varepsilon }}^{p}-{\varepsilon^{\prime }}_{N}}{{s^{\prime }}_{N}}\right)}^{2}\right]$$

It is in the same form as *A*, in which *D*_N_ denotes the fraction of second phase particles with nucleation potential. ${\varepsilon^{\prime }}_{N}$ and ${s^{\prime }}_{N}$ are the average strain and its standard deviation related to the nucleation of the second phase.

3. The coupling effect of tension damage and shear damage

Considering the influence of void nucleation under shear deformation on tension damage and under tension deformation on shear damage during tension–shear deformation, Equations (9) and (14) are modified as follows:

(17) $${\dot{f}}_{g}=(1-f^{**}){\dot{\varepsilon }}_{\nu }^{p}$$

(18) $${\dot{D}}_{shear}=\frac{3}{2}{\left(\frac{6}{\pi }\right)}^{1/3}f^{**1/3}{\bar{\varepsilon }}^{p}{\dot{\bar{\varepsilon }}}^{p}$$

where $f^{**}$ is tension–shear damage unified via the stress state characterization equation, and the formula is given as

(19) $$f^{**}=(1-G_{\left(\sigma \right)})f+G_{\left(\sigma \right)}D$$

Stress triaxiality and the Lode parameter are coupled by the stress state equation of $G_{\left(\sigma \right)}$.

**Figure 2:**
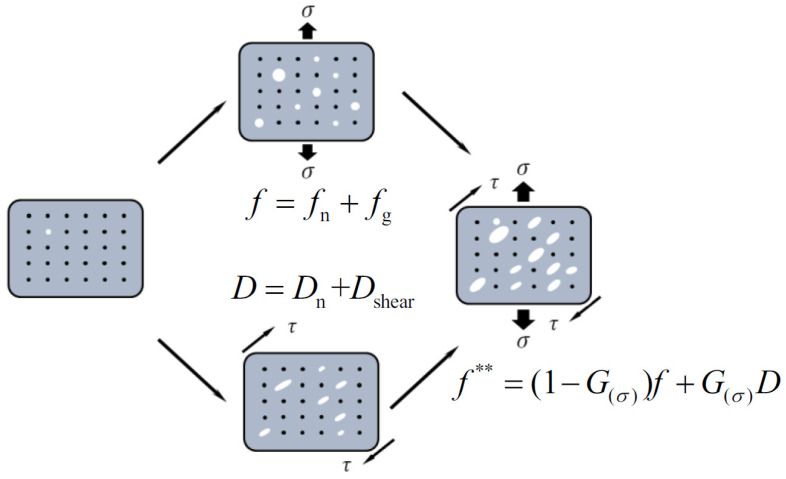
The evolution of damage under a general stress state.

As shown in Figure 2, since the nucleation and growth of voids is different at a compressive stress state (η<0) and a tension stress state (η>0), the asymmetric stress state function is employed. Namely, in the case of low stress triaxiality, there is no initiation of voids, and the closure effect of voids even takes place, so the volume fraction of voids decreases. Under high stress triaxiality, the voids initiate and grow rapidly. Therefore, the stress state function is asymmetric with respect to stress triaxiality. Through the adjustment of the stress state function, the equivalent plastic strain affects the growth of shear voids, and the volumetric strain affects the growth of tension voids.

4. Coalescence criterion

The void coalescence criterion proposed by Malcher et al. [20] is used in this paper, as given by Equation (20). Considering the critical value of tension damage and shear damage, the normalized equivalent damage is used in the void coalescence criterion, in which ($1+\frac{f_{c}}{D_{c}}$) is used as the accelerator parameter of damage to characterize the accelerated void coalescence under tension–shear deformation.

(20) $$E_{fD}=\left\{\begin{array}{l} \frac{D}{D_{c}}\\ \left(1+\frac{f_{c}}{D_{c}})(\frac{f}{f_{c}}+\frac{D}{D_{c}}\right)\\ \frac{f}{f_{c}} \end{array}\right.\begin{array}{c} G_{\left(\sigma \right)}=1\\ \;\;0<G_{\left(\sigma \right)}<1\\ G_{\left(\sigma \right)}=0 \end{array}$$

where $f_{c}$ and $D_{c}$ are the corresponding tension damage and shear damage thresholds.

### 2.3. Constitutive Model

(1) Yield equation with considering damage

In order to achieve the effective prediction of damage and fractures under high stress triaxiality and low stress triaxiality, some reasonable improvements are put forward to modify the GTN model with a newly proposed stress state characterization equation coupling the Lode angle and stress triaxiality, i.e.,

(21) $$\Phi ={\left(\frac{\sigma_{\mathrm{eq}}}{{\bar{\sigma }}_{M}}\right)}^{2}+2q_{1}f^{**}\cos h(-\frac{3q_{2}\sigma_{H}}{2{\bar{\sigma }}_{M}})-1-q_{3}f^{**2}=0$$

Obviously, in a uniaxial tensile stress state with high stress triaxiality, $G_{\left(\sigma \right)}=0$. Thus, $f^{**}=f$. The yield equation degenerates into the original GTN model with tension damage related to void nucleation and growth. Under a pure shear stress state with low stress triaxiality, $G_{\left(\sigma \right)}=1$. Thus, $f^{**}=D$. In the pure shear state, $\sigma_{H}=0$. The yield equation becomes:

(22) $$\Phi ={\left(\frac{\sigma_{\mathrm{eq}}}{{\bar{\sigma }}_{M}}\right)}^{2}+2q_{1}D-1-q_{3}D^{2}={\left(\frac{\sigma_{\mathrm{eq}}}{{\bar{\sigma }}_{M}}\right)}^{2}-{\left(1-q_{1}D\right)}^{2}=0$$

If we take $q_{3}={\left(q_{1}\right)}^{2}$, the above equation turns into:

(23) $$\frac{\sigma_{\mathrm{eq}}}{1-q_{1}D}={\bar{\sigma }}_{M}$$

It is equivalent to the Lemaitre damage model. Under combined stress states (axisymmetric stress state + pure shear stress state), the proportion of tension damage and shear damage can be adjusted by the stress state equation. Therefore, the above modification is reasonable.

(2) Flow rule

In this contribution, the associated flow rule is adopted, in which the yield function is defined as the potential function, so the plastic strain rate is:

(24) $${\dot{\varepsilon }}^{p}=\dot{\lambda }\frac{\partial \Phi }{\partial \sigma }={\dot{\varepsilon }}_{d}^{p}+{\dot{\varepsilon }}_{\nu }^{p}=\dot{\lambda }(\frac{3}{{\bar{\sigma }}_{M}^{2}}S+\frac{f^{**}q_{1}q_{2}}{{\bar{\sigma }}_{M}}sinh(-\frac{3q_{2}\sigma_{H}}{2{\bar{\sigma }}_{M}})I)$$

where $\dot{\lambda }$ is the plastic multiplier and **I** is the unit tensor. ${\dot{\varepsilon }}_{d}^{p}$ is the plastic strain rate caused by the stress deviator, and ${\dot{\varepsilon }}_{\nu }^{p}$ is the plastic volumetric strain rate. Then, the equivalent plastic strain rate is as follows:

(25) $${\dot{\bar{\varepsilon }}}^{p}=\sqrt{\frac{2}{3}{\dot{\varepsilon }}^{p}:{\dot{\varepsilon }}^{p}}=\dot{\lambda }\sqrt{\frac{2}{3}\left\{\frac{9}{{\bar{\sigma }}_{M}^{4}}S:S+{\left[\frac{f^{**}q_{1}q_{2}}{{\bar{\sigma }}_{M}}sinh(-\frac{3q_{2}\sigma_{H}}{2{\bar{\sigma }}_{M}})I\right]}^{2}\right\}}$$

(3) Plastic behavior of the matrix

Since the anisotropy of annealed 2219 aluminum alloy is not significant, the isotropic hardening rule is used to describe the plastic deformation behavior of annealed 2219 aluminum alloy. Based on the Swift model [26],

(26) $${\bar{\sigma }}_{M}=K{\left(\varepsilon_{0}+{\bar{\varepsilon }}_{M}^{P}\right)}^{n}$$

where ${\bar{\varepsilon }}_{M}^{P}$ is the equivalent plastic strain of the matrix, which is determined according to the conservation of the material’s plastic work. Considering the influence of damage, there is:

(27) $${\bar{\sigma }}_{M}{\dot{\bar{\varepsilon }}}^{P}=\frac{\sigma :{\dot{\varepsilon }}^{p}}{1-f^{**}}$$

### 2.4. Determination and Verification of Model Parameters

**Figure 3:**
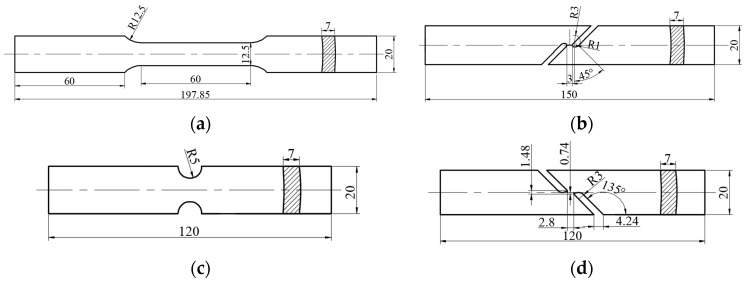
Dimensions of the specimens taken from the 2219 aluminum alloy tubular billet: (**a**) uniaxial tension; (**b**) shear; (**c**) Nakazima test; (**d**) tension–shear.

In this paper, the inverse finite element method is employed to calibrate the damage parameters. Four kinds of mechanical tests are conducted to determinate model parameters. Figure 3 exhibits the dimensions of 2219 aluminum alloy tubular billet specimens. The mechanical property parameters of the annealed 2219 aluminum alloy tubular billet specimens are shown in Table 2. The values of elastic modulus (*E*) and Poisson’s ratio (*ν*) are 68 GPa and 0.3, respectively. The Swift model of annealed 2219 aluminum alloy is fitted as

(28) $${\bar{\sigma }}_{M}=291.8{\left(0.0001+{\bar{\varepsilon }}_{M}^{P}\right)}^{0.219}$$

**Figure 4:**
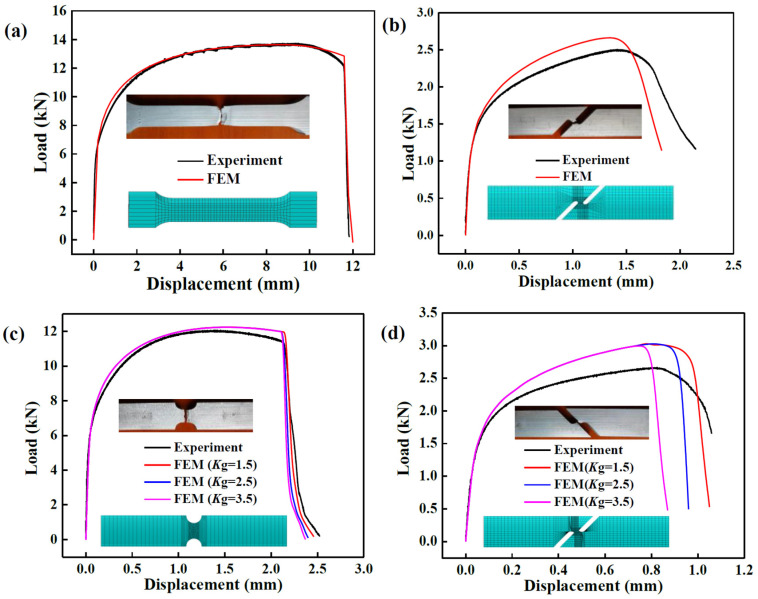
Load displacement curves of annealed 2219 aluminum alloy tubular billet specimens obtained by simulation and experiment: (**a**) uniaxial tension; (**b**) shear; (**c**) Nakazima test; (**d**) tension–shear.

The load displacement curves obtained by finite element (FE) simulation and experiments are compared to verify the accuracy of the parameters, as shown in Figure 4. In the FEM, the mesh sizes of the uniaxial tension specimen are 1.75 mm in RD (thickness direction), 1.71 mm in CD (width direction) and 1.56 mm in AD (length direction). For the shear deformation specimen, the mesh sizes of the middle refinement zone are 0.3 mm in RD and 0.25 mm in AD and CD. For the Nakazima test specimen, the mesh sizes of the middle refinement zone are 0.5 mm in RD and 0.25 mm in AD and CD. As for the tension–shear test specimen, the mesh sizes of the middle refinement zone are 0.5 mm in RD and 0.3 mm in AD and CD. The tension speed is 2 mm/s for the uniaxial tension and Nakazima tests and 1 mm/s for the shear and tension–shear tests.

It can be seen that the gap between the simulation and experiment load is small for the uniaxial tension (Figure 4a) and Nakazima tests (Figure 4c), while it is a little larger for the shear deformation (Figure 4b) and tension–shear (Figure 4d) tests. Due to the arc shape of the specimens cut from the tubular billet, there are sharp corners in the middle connection area of the Nakazima test and tension–shear specimens, resulting in rapid instability during shearing. On the other hand, mesh size also has a significant impact on the simulated loads on account of small shear deformation zones. Thus, there is a lager difference between the simulated load and experimental load in the Nakazima test and tension–shear test. On the whole, the largest simulation and experiment load deviation is less than 15%, which is the same as the result obtained by Wu et al. [13]. In order to ensure the reliability of FEM, consistent fracture displacement between the simulation and the experiment is used to determine the model parameters. The damage parameters of the annealed 2219 aluminum alloy tubular billet are shown in Table 3. In addition, the values recommended in Tvergaard and Needleman’s research [15] are adopted, i.e., *q*_1_ = 1.5, *q*_2_ = 1, *q*_3_ = (*q*_1_)^2^ = 2.25.

**Figure 5:**
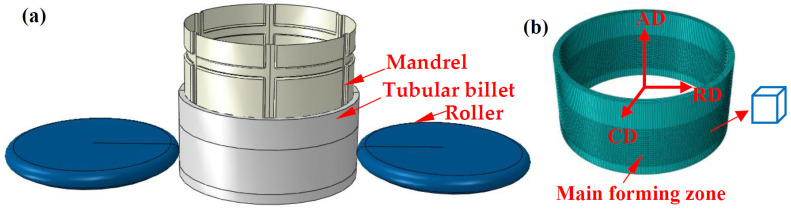
FEM of flow forming of tubular parts with LTIRs: (**a**) FEM, (**b**) local mesh refinement.

The FE simulation of flow forming of tubular parts with LTIRs is carried out using the Abaqus/Explicit platform. Therein, the numerical implementation of the modified GTN model was based on a semi-implicit return-mapping algorithm in the user-defined VUMAT code, which was introduced by Li et al. [11]. The corresponding finite element model (FEM) is shown in Figure 5. The wall thickness, length and internal diameter of the tubular billet are 7 mm, 100 mm and 200 mm, respectively. The diameter and tip radius of the rollers are 200 mm and 10 mm. The diameter of the mandrel is 199.7 mm and the fillet radius of the rib grooves is 2 mm. The width and depth of the longitudinal and transverse rib grooves are 10 mm and 4 mm, respectively. The six transverse rib grooves are evenly distributed along the circumferential direction. The intervals between the longitudinal rib grooves are 25 mm and 50 mm, respectively. The tubular billet is divided into an unformed area (36 mm), main forming area (53 mm) and fixed area (12 mm), in which the end of the fixed area is set with a coupling constraint with the mandrel. The tubular billet is defined as a three-dimensional deformable body, while the mandrel and rollers are set as a three-dimensional discrete rigid body and analytical rigid body, respectively.

During spinning, the tubular billet rotates with the mandrel at a speed of 80 rev/min. The two rollers first move along the radial direction (RD) until they reach the set wall thinning rate (28.6%, 42.9%, 52.9% and 57.1%, respectively). Then, they move along the axial direction (AD) with the set feed ratio (1 mm/rev and 2 mm/rev, respectively) to form the ribs. In order to improve the simulation efficiency and accuracy, local mesh refinement is applied in the main forming area. The mesh sizes in the main forming area of the tubular billet along the AD, circumferential direction (CD) and RD are 1.76 mm, 1.88 mm and 1.75 mm, respectively. The mesh element type is set as an eight-node reduced integration brick element (C3D8R). The mesh quantity of the tubular billet is 45,824 and the mass-scaling factor is 5000. The friction coefficient between the mandrel and the tubular billet is 0.01, while that between the rollers and the tubular billet is 0.1. Enhanced hourglass control is used during the simulation.

**Figure 6:**
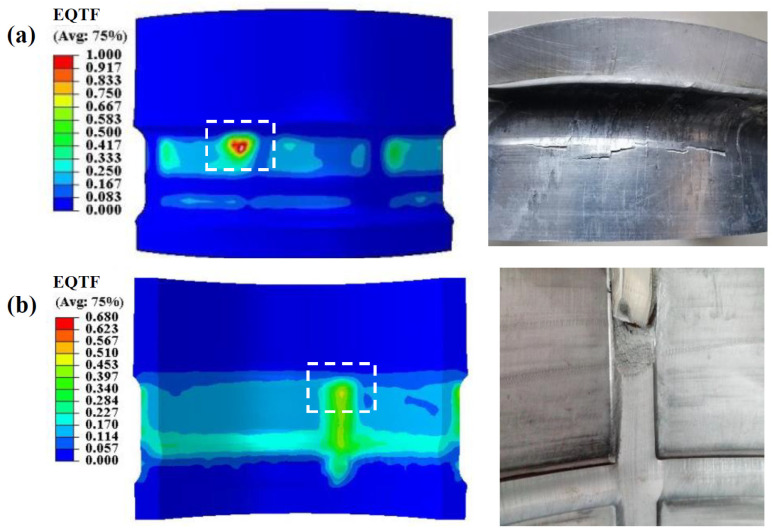
The comparison of damage and fracture between flow forming simulation and experiment results: (**a**) at NRZ; (**b**) at LIR.

In order to verify the reliability of the modified GTN model in the damage prediction of tubular parts with LTIRs, the simulation results are compared with experimental results. As shown in Figure 6a, when flow forming is carried out under a wall thickness reduction rate of 57.1% and a feed ratio of 2 mm/rev, a large amount of built-up material occurs in front of the rollers at the axial feeding stage, and cracks occur at the NRZ. Meanwhile, the total equivalent damage (EQTF) predicted by FE simulation on the surface of the NRZ exceeds the damage threshold, indicating that the FEM is reliable, as shown in the white dashed box. In addition, Figure 6b compares the experimental and simulated damage and fracture defects on the inner surface of LIR under *l* = 50 mm. Since the simulation will terminate at an extremely large Δ*T*, the wall thinning rate in the simulation is slightly smaller than that in the experiment. The details will be explained in Section 3.2.1. A fracture occurs on the inner surface of LIR in the experiment, while the damage value at the position (in the white dashed box) corresponding to the experiment is the highest. Although the predicted total equivalent damage to the inner surface of LIR has not reached the damage threshold, the risk of fracture is the highest, suggesting that the simulation results are acceptable.

## 3. Results and Discussion

### 3.1. Fracture Behavior of Tubular Parts with LTIRs

#### 3.1.1. Fracture at NRZ

**Figure 7:**
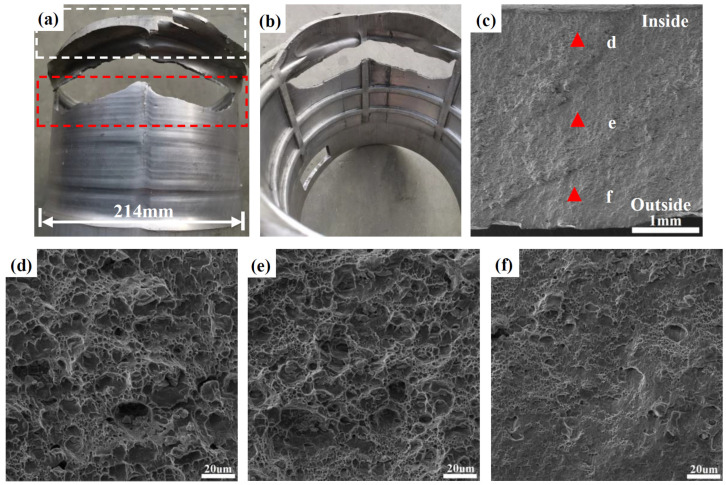
Fracture location and fracture morphology at the NRZ: (**a**) the outer surface; (**b**) the inner surface; (**c**) the overall morphology; (**d**) near the inner surface; (**e**) at the center; (**f**) near the outer surface.

The deformation mode of the NRZ was approximately plane strain deformation, i.e., axial tension and radial compression. Thus, shear strain was large at the NRZ. Moreover, material build-up easily occurred in front of the rollers due to the large amounts of bulged material at large Δ*T*, resulting in a strong tension/stretching effect at the NRZ. Therefore, fracture defects are likely to occur. As shown in Figure 7a,b, fractures occurred at the NRZ under a wall thinning rate of 52.9%, as shown in the red dotted box. The white dotted box denotes the material build-up in front of the rollers. Some material on the outer layer of the formed part is sheared off, visible as trimming in the white dotted box.

Figure 7c–f show the morphology of the fractures at the NRZ observed along the RD. The fraction of dimples on the inside and middle layers was larger than that on the outside layer. Among them, the dimples on the inside and middle layers of the NRZ show an equiaxed shape. However, the dimples are smaller and shallower on the outside layer, and there are almost no dimples near the outer surface of the formed part. This implies that tension-dominated deformation caused the fracture emerging on the inside of the NRZ, while degenerated material ductility along with a sharp reduction in the number of dimples on the outside is due to strong shear deformation and work hardening.

#### 3.1.2. Fracture at LIR

**Figure 8:**
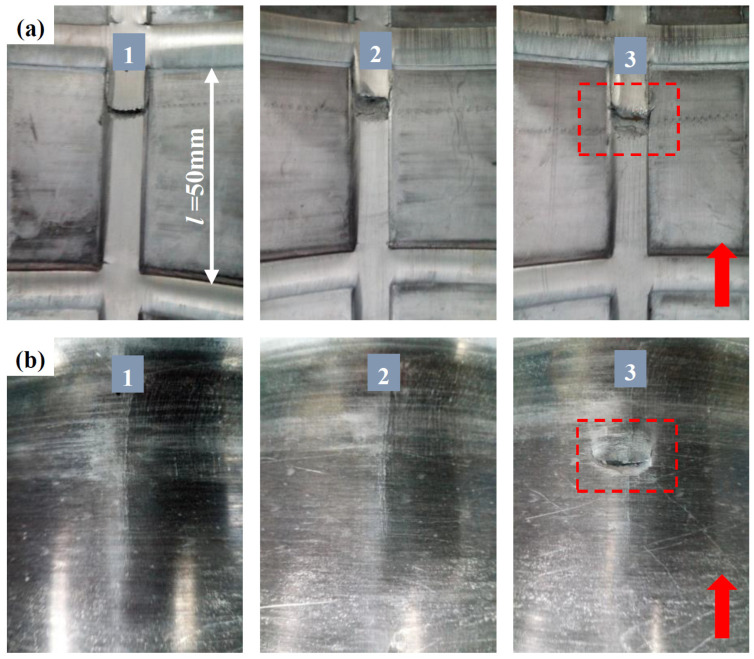
Fractured LIRs at *l =* 50 mm: (**a**) the inner surface; (**b**) the outer surface.

The geometric parameters of the mandrel affect not only the formability of inner ribs, but also the fracture behavior of the LIR. At a large *l*, the bulged material in front of the rollers increases, which will result in easy fractures on LIRs. In Figure 8, fractures occur on the inner and outer surfaces of some LIRs at *l* = 50 mm and a wall thickness reduction rate of 42.9%. The red arrows in the figure denote the motion direction of the rollers (along the AD). The fracture locations suggest that fractures occur at the end of LIRs away from the first formed TIR. For LIRs No. 1 and No. 2, fractures only occur on the inner surface. However, both the inner and outer surfaces of LIR No. 3 are cracked. Moreover, it can be seen from the inner surface of the LIR that there is a certain necking at the fracture location, and the fractured surface is inclined to the RD, which is similar to the fracture morphology of uniaxial tension. A concave deformation is present on the fractured surface of LIR No. 3.

**Figure 9:**
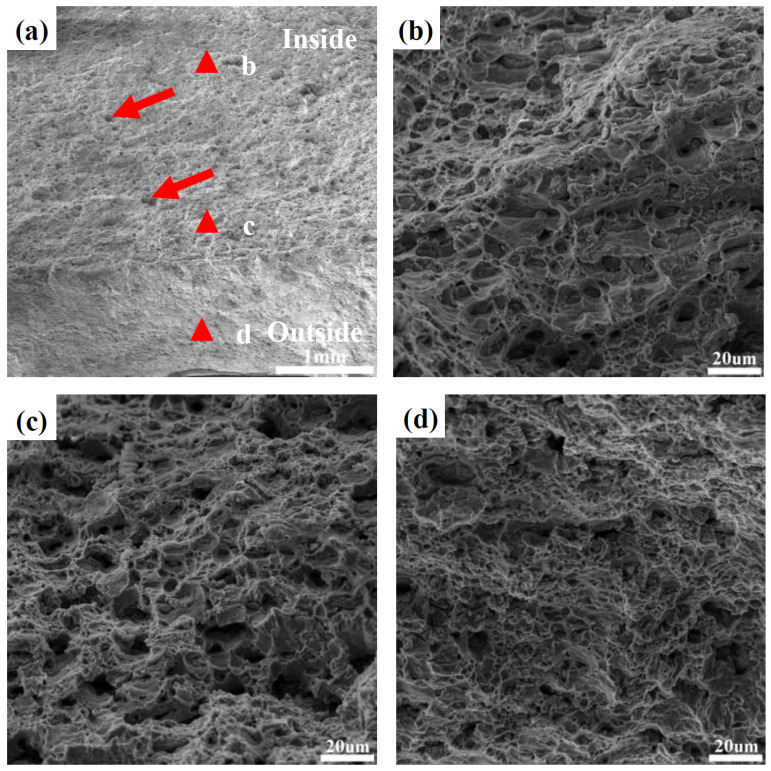
Fracture morphology of a fractured LIR: (**a**) the overall morphology; (**b**) near the inner surface; (**c**) at the center; (**d**) near the outer surface.

Figure 9 shows the fracture morphology of a fractured LIR. Some obvious voids are spread on the fractured surface, as indicated by the red arrows in the SEM graph at low magnification (Figure 9a). Macroscopically, the V-shaped LIR section is similar to shear fractures, but no elongated shearing dimples can be found on the fractured surface. On the contrary, the large number of equiaxial dimples implies that fractures on LIRs is tension-dominated.

The dimples on the inside of the LIR are large and deep, and some residual broken coarse second phase particles are at the bottom of the dimples. In addition, some interconnected dimples can be seen in the graph. The number of dimples on the middle and outside layers of LIR is significantly lower than that on the inside layer, and their depth is also shallower than that on the inside layer of the LIR. This implies that the damage level and generation priority may be different between the inside and outside layers of LIRs. In addition, although both the NRZ and the inside of the LIR show the characteristics of tension fractures, the number, size and depth of dimples are obviously different, which may be related to the different deformation modes of the NRZ and the LIR.

#### 3.1.3. Fracture at TIR

**Figure 10:**
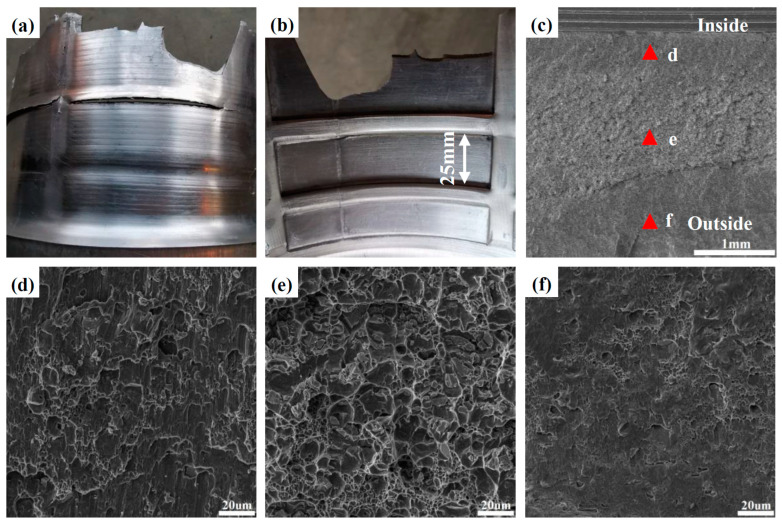
Fracture morphology at the interface of the TIR and NRZ: (a) the outer surface; (b) the inner surface; (**c**) the overall morphology; (**d**) near the inner surface; (**e**) at the center; (**f**) near the outer surface.

Figure 10 shows a fracture occurring at the interface between the TIR and the NRZ under a wall thinning rate of 57.1%. Due to the large Δ*T*, the groove of the TIR is almost fully filled, and the remnant wall thickness is small at the NRZ after flow forming. The radial tension deformation is strong when material fills in the TIR groove along the RD, which plays an important role in the occurrence of fractures at the interface. Moreover, TIR grooves have a strong shear effect on the inflow material. The smaller the draft angle of TIR grooves, the stronger the shear effect. In addition, stress concentration is likely to occur at the interface, accelerating the propagation of fractures.

Figure 10c–f show the fracture morphology at the interface. In Figure 10c, there is an obvious boundary between the inside and outside layers. Many voids occur on the inside layer, as indicated by the red arrows. The high magnification enabled by SEM shows that there are obvious elongated shearing dimples on the inside of the fractured surface. Thus, shear deformation plays an important role in fractures of the inside interface. The outside interface is similar to the outside layer of the NRZ, with small and shallow dimples. It may be related to the strong work hardening effect aggravated by built-up material that fills the TIR groove during the subsequent formation of the TIR. In addition, a large number of equiaxed dimples are distributed in the middle of the interface, indicating that interface fractures are also affected by tension deformation. Therefore, the different fracture morphology suggests that the fracture mode at the interface between the TIR and the NRZ is complex.

### 3.2. Fracture Mechanisms of Tubular Parts with LTIRs

#### 3.2.1. Damage Evolution at NRZ

**Figure 11:**
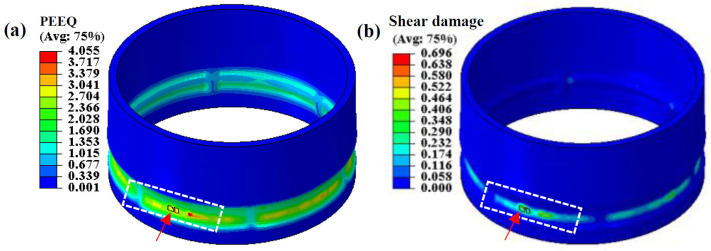
Fracture at NRZ under large Δ*T*: (**a**) PEEQ; (**b**) shear damage.

In order to study the damage and fracture mechanism of the NRZ, damage distribution and evolution at the NRZ under different wall thickness reduction rates were analyzed. Figure 11 shows the distribution of PEEQ and shear damage at a wall thinning rate of 57.1%. Due to the strong shear effect attributed to the large amount of built-up material, the shear damage to the outer surface of the NRZ is great, which is consistent with the experimental finding that some materials are sheared off under large Δ*T*. The position pointed by the red arrow indicates that the material is removed after reaching the damage threshold. At a large deformation, the damage threshold of spinning is higher than that determined by simple deformation (uniaxial tension, shear, etc.), which has been confirmed by many scholars. For example, Zhan et al. [27], Martínez-Donaire et al. [28] and Seong et al. [29] gave a detailed explanation for why the fracture-forming limit in complex deformation is higher than that in simple deformation. The main reasons are as follows: first, the stress–strain gradient delays or inhibits local necking in this non-proportional incremental forming. Second, damage accumulation is discontinuous in a reciprocated loading process. The damage accumulation time of a single unit during cyclic local loading is very short. Namely when the forming die approaches and passes by the unit, damage begins to accumulate, but damage does not increase when the forming die is away. The third is that the strong non-proportional loading of complex deformation leads to changes in the strain path at the fracture location. During flow forming, the deformation area undergoes repeated loading and unloading under the reciprocal motion of rollers and the stress state is complex. Therefore, this paper mainly studies the multi-mode damage and fracture mechanisms of tubular parts with LTIRs via FE simulation.

**Figure 12:**
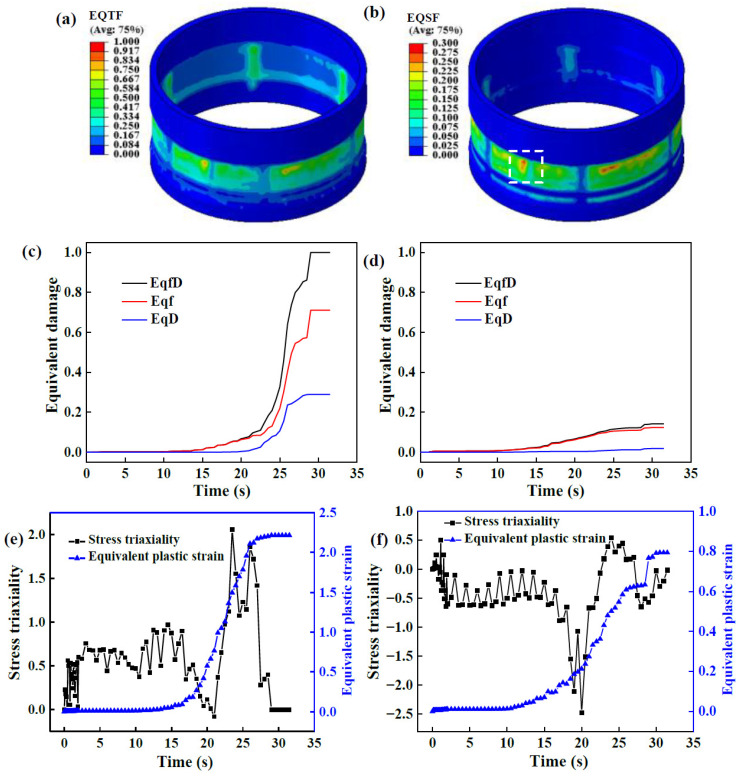
The distribution and evolution of damage, stress triaxiality and PEEQ on the NRZ: (**a**) EQTF; (**b**) EQSF; (**c**) element on the outer surface; (**d**) element on the inner surface; (**e**) element on the outer surface; (**f**) element on the inner surface.

Figure 12 shows the damage distribution and evolution at the NRZ when the wall thickness reduction rate is 28.6% under a large *l*. The damage value at the outer surface of the NRZ is the highest, and shear damage accounts for a large proportion of it, as shown in Figure 12a,b. This is mainly because, on the one hand, the outer surface of the NRZ is subjected to large shear strain, resulting in shear damage. On the other hand, the built-up material in front of the rollers at the NRZ increases with increasing *l*, as does the tensile strain in the forming area. Therefore, the damage value at the outer surface of the NRZ far away from the first TIR is large.

Figure 12c shows the outer surface damage evolution of the NRZ at the point with maximum damage. The damage value is 0 when the roller is far away. As the roller approaches this point, tension damage rises due to bulged material on the outer surface of the NRZ. Moreover, shear damage increases rapidly under the shear effect of the rollers. When the rollers pass by, the built-up material in front of the rollers increases the tension effect on the formed area, resulting in a rapid increase in tension damage.

It should be noted that the threshold of shear damage is much higher than that of tension damage. Consequently, although the equivalent shear damage (EQSF) is lower than the equivalent tension damage (EQVF), the increased void volume fraction caused by shear damage (*D*) accounts for a large proportion of the total volume fraction of voids ($f^{**}$). Thus, total damage increases rapidly due to the coupled tension–shear effect in the modified GTN model. In addition, the damage evolution of the corresponding element on the inner surface of the NRZ is shown in Figure 12d. The damage to the inner surface also increases with the approaching of the rollers. However, the damage value on the inner surface is lower compared with that on the outer surface, especially for shear damage, which is almost 0.

Figure 12e,f show the evolution of stress triaxiality and PEEQ of the outside and inside NRZ elements. The stress triaxiality of most outside elements is greater than 0. As the roller passes by, stress triaxiality reaches the maximum. When the roller moves far away, stress triaxiality decreases to 0. Since a high stress triaxiality is conducive to the nucleation and growth of voids, it is beneficial to the rapid accumulation of damage. The larger damage value on the outer surface of the middle NRZ is shown in Figure 12c. Otherwise, damage accumulation is inhibited by low stress triaxiality, e.g., on the inside of NRZ under compression deformation. However, when the rollers pass through the element, stress triaxiality is greater than 0 due to the tension effect of the unformed area. In addition, PEEQ of the inside and outside elements gradually increases as the rollers approach. Finally, it falls to a stable value. PEEQ on the outer surface is significantly greater than that on the inner surface. According to the law of void nucleation, an increase in PEEQ is beneficial to damage accumulation.

#### 3.2.2. Damage Evolution at LIR

**Figure 13:**
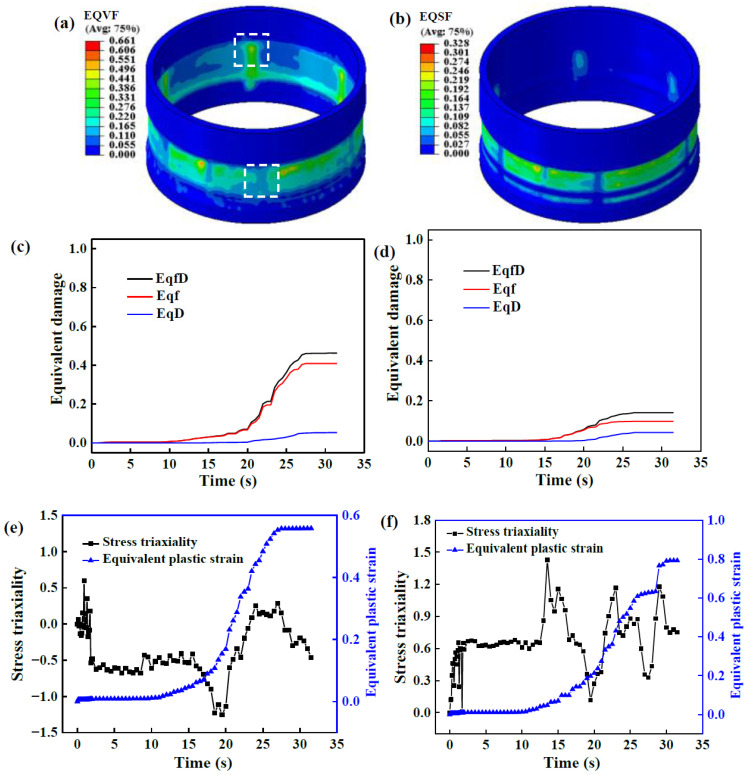
The distribution and evolution of damage, stress triaxiality and PEEQ at the LIR: (**a**) EQVF; (**b**) EQSF; (**c**) element on the inner surface; (**d**) element on the outer surface; (**e**) element on the inner surface; (**f**) element on the outer surface.

Figure 13a,b show the equivalent tension and shear damage at the LIR, respectively. Tension and shear damage at the inner surface of the LIR are higher than those at the outer surface. Therefore, tension damage is much greater than shear damage, implying that tension damage is responsible for fractures on the LIR under a large *l*. However, shear damage accelerates tension damage since it promotes a rapid increase in void volume fraction.

During the flow forming of the LIR, the material’s axial movement is not hindered owing to the free front end of the LIR groove, which promotes the axial extension of the LIR. Under a large *l*, the thinned material on the outer layer of the NRZ cannot fill the TIR grooves in time, and it mainly flows along the AD. However, the material at the LIR mainly fills in the LIR groove along the RD. Thus, in the same section, perpendicular to the AD, the axial movement speed of the material at the NRZ is greater than that at the LIR. Consequently, the axial strain gradient along with additional tension stress comes into being, which increases with increasing *l*. In addition, the unformed area has an axial tension effect on the formed LIR. Therefore, tension damage easily occurs on the LIR.

The equivalent damage evolution of the inner and outer surface elements on the LIR with the maximum damage value is shown in Figure 13c,d. With an increase in forming time, tension damage increases quickly, resulting in a rapid increase in total equivalent damage. The proportion of shear damage to the outer surface is higher than that to the inner surface. In addition, for the inner surface of the LIR, the surface damage value of the LIR at the intersection of the LTIR and away from the first TIR is higher than in the other positions. The damage value of the latter is higher than that of the former, which is consistent with the experimental results described in Section 3.1.2., i.e., fractures occur on the LIR and are located away from the end of the first TIR.

Figure 13e,f show the evolution of stress triaxiality and PEEQ of the inner and outer surface elements. Similar to the NRZ, the stress triaxiality of the inner surface of the LIR at the forming stage is greater than 0, while at other stages it is mostly less than 0. On the outer surface, stress triaxiality is almost greater than 0. However, different from the NRZ, PEEQ on the outer surface of the LIR is much smaller than that on the outer surface of the NRZ, while the difference between them on the inner surfaces is small. Because the growth of tension and shear damage is related to PEEQ, the difference in PEEQ between the NRZ and LIR is one of the main reasons for their different damage evolution laws. On the other hand, because shear damage to the inner surface of the NRZ is almost equal to 0, shear damage to the inner surface of the LIR contributes greatly to total damage. Hence, the difference between shear damage to the inner surface of the NRZ and LIR is another main reason for their different damage evolution laws.

**Figure 14:**
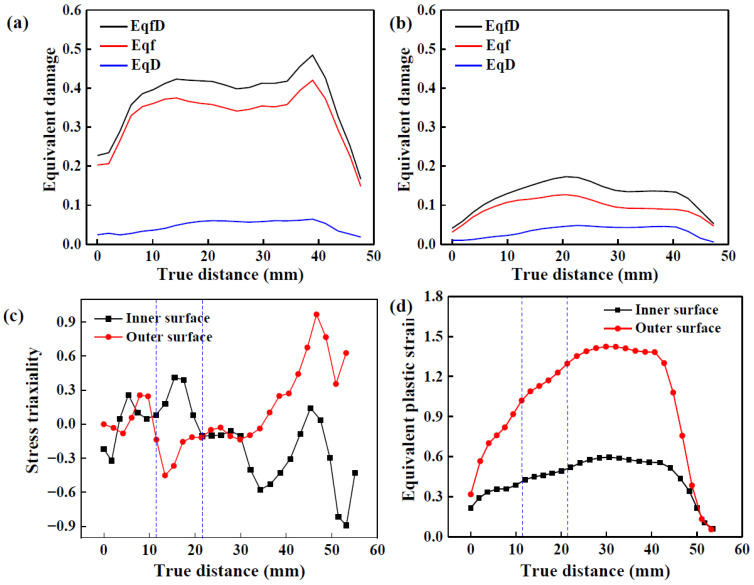
Equivalent damage, stress triaxiality and PEEQ on the inner and outer surface of the LIR: (**a**) equivalent damage to the inner surface; (**b**) equivalent damage to the outer surface; (**c**) stress triaxiality; (**d**) PEEQ.

Figure 14a,b show the damage evolution on the inner and outer surfaces of the LIR along the AD. For the inner surface of the LIR, the damage value away from the first TIR is the highest. However, the damage value at the intersection of the LTIR is slightly higher on the outer surface. On the whole, the damage value of the inner surface is obviously higher than that of the outer surface, in which tension damage plays a major role. As the built-up material hinders the axial movement of the rollers, the formed area is subjected to additional tension strain in the subsequent deformation. The tensile stress in the deformed area away from the forming area is relatively uniform due to its stiffened structure, as its LIRs are conjunct with the NRZ, but the area formed near the forming area suffers from a more intense tension effect. Moreover, a large amount of built-up material brings in a strong shear effect in the forming area. Therefore, the surface damage value of the LIRs near the forming area is high. In fact, due to the large fillet radius of the rollers, the contact area between the rollers and the material is large. At a large Δ*T*, more material is accumulated in front of the rollers, resulting in greater tensile stress. Since the inner layer of the LIR is a free surface, the deformation mode is different from that of NRZ. The shear damage at low stress triaxiality also promotes total damage accumulation.

Figure 14c,d show the distribution of stress triaxiality and PEEQ on the inner and outer surfaces of the LIR. The area enclosed by the blue dotted line is the corresponding position at the intersection of the LTIR. At the current moment, stress triaxiality at the inner surface is greater than 0, while that at the outer surface is less than 0. As for the LIR, stress triaxiality at the inner surface of the formed area is less than 0, while that in the forming area is greater than 0, resulting in a rapid increase in damage to the inner surface. At the position in front of the rollers, the stress triaxiality of the inner surface of the LIR is less than 0, but that on the outer surface of the LIR is greater than 0. This may be caused by the bulge deformation of the material. PEEQ on the LIR is the largest, as shown in Figure 14d, which is conducive to damage accumulation, resulting in a higher damage value to the LIR.

#### 3.2.3. Damage Evolution at the Interface of TIR

**Figure 15:**
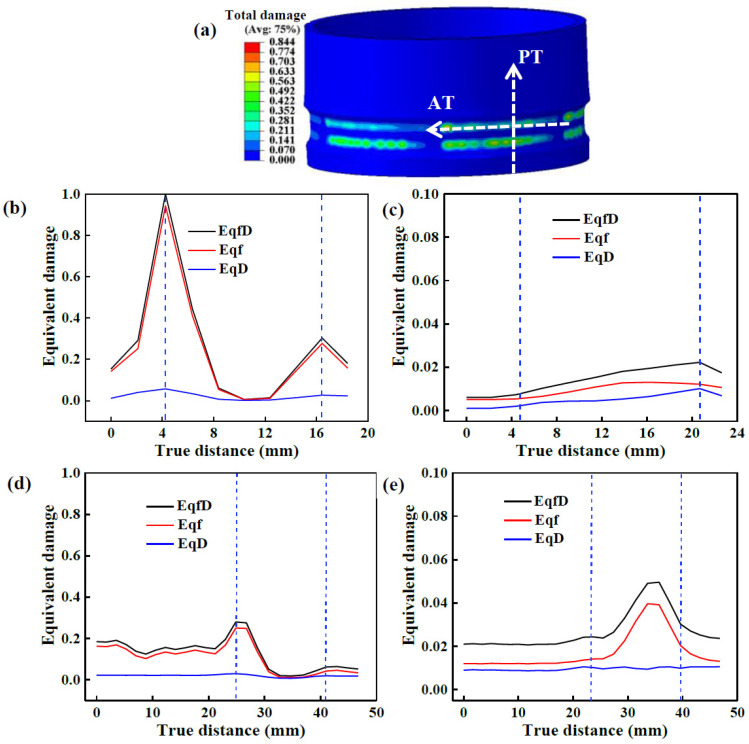
Damage variation along different paths: (**a**) the two paths; (**b**) outer surface damage along the PT; (**c**) inner surface damage along the PT; (**d**) outer surface damage along the AT; (**e**) inner surface damage along the AT.

Under a large wall thickness reduction rate, fractures is likely to occur at the interface between the TIR and NRZ. Figure 15 shows the damage distribution along the paths perpendicular to the TIR (PT) and along the TIR (AT), as shown in Figure 15a. The tension damage threshold exceeds the fracture threshold of simple deformation due to the large Δ*T*. Thus, the damage value at the moment of fracture is used as the damage threshold. In Figure 15b, the damage value of the outer surface at the interface between the TIR groove and the NRZ is the highest, while that at the TIR groove is the lowest. Therein, the damage value of the outer surface at the start of the TIR groove filling stage is higher than that at the end of the filling stage. In addition, the damage value at the outer surface of the TIR is the lowest. However, the damage variation at the inner surface is the opposite, as shown in Figure 15c. During the filling stage, damage increases gradually with the movement of the rollers. Excluding the influence of mesh size in FEM, the interface between the TIR and NRZ can be judged in terms of the obvious sudden change in damage value.

When the TIR groove is about to be filled, the rollers will move from the NRZ to the TIR groove. The deformation of the material on the outer layer is larger than that of the inner layer, so the damage value of the outer surface is higher than that of the inner surface. At the filling stage, the material at the interface between the TIR and NRZ is subjected to shear deformation applied by the TIR groove and to radial tension deformation applied by the filled material, resulting in a higher damage value at the outer surface. The damage value at the inner layer is low because the inner surface of the TIR is a free surface, and the material moves along the AD with little deformation resistance.

When the filling stage of the TIR groove is about to be completed, there is less built-up material in front of the roller. Although the rib groove interface imposes shearing on the material, there is no tension deformation applied by the filled material, so the damage to the outer surface here is lower than that to the outer surface at the onset of LTIR forming. The damage value is higher than that on the inner surface at the initial filling stage, which results from the TIR groove’s hindering effect on the axial movement of the material and the shearing of the groove interface on the material.

Figure 15d,e show the damage distribution on the outer and inner surfaces of the NRZ along the AT, respectively. The effect of the LIR groove on damage evolution is similar to that of the TIR groove, i.e., the damage to the outer surface is higher than that to the inner surface. For the outer surface, the damage to the interface where the inner rib begins to form is higher than that to the formed interface, while the variation in damage to the inner surface is the opposite. Therefore, it is further confirmed that the shearing applied by the rib groove on the filling material and the tension effect of the filled material on the material at the rib groove make the interface between the rib groove and NRZ prone to fractures, especially under a large Δ*T*.

**Figure 16:**
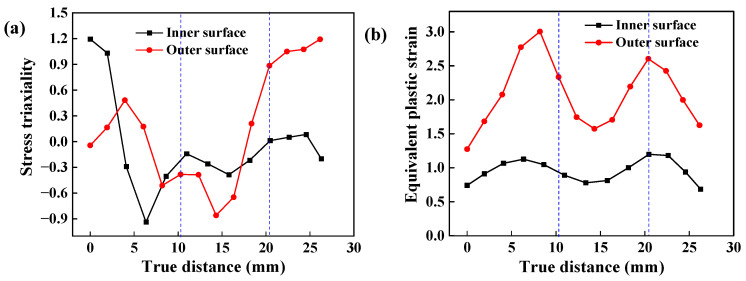
Stress triaxiality and PEEQ at the inner and outer surface along the PT path: (**a**) stress triaxiality; (**b**) PEEQ.

Figure 16 shows the distribution of stress triaxiality and PEEQ at the inner and outer surfaces measured along the PT path, in which the front end of the PT path is the forming area. The area enclosed by the blue dotted line is the corresponding position of the TIR, with a negative stress triaxiality. Stress triaxiality at the outer surface of the NRZ is positive, while that on most inner surface is negative. This suggests that the deformation modes at the TIR and NRZ are different, as are those on the inner and outer surfaces of the TIR. Tension deformation plays a major role on the outer surface near the interface between the TIR and NRZ, while the inner surface mainly suffers from shear deformation at low stress triaxiality. It confirms that tension–shear deformation is dominant at the interface between the TIR and NRZ. Moreover, there is a large difference in PEEQ at the interface between the NRZ and TIR, as shown in Figure 16b, so it is easy to produce high damage here, thus resulting in the occurrence of fractures.

## 4. Conclusions and Prospects

In this paper, a new strategy for coupling tension damage and shear damage was used in a modified GTN model in which the damage variables were unified by means of a new asymmetric stress state function. The tension-dominated and shear-accelerated damage evolution of thin-walled tubular parts with LTIRs was well predicted, and their multi-mode fracture mechanisms were revealed. The following conclusions can be made:

(1) The material sheared off from the outer surface of the tubular part comes from the build-up of material in front of the rollers under a large wall thickness reduction rate. Tension-dominated deformation causes fractures to emerge at the inner layer of the NRZ, while strong shear deformation and work hardening are the causes of a fractured surface with few dimples at the outer layer;
(2) Axial tension deformation is mainly responsible for the fracture of LIRs at large TIR intervals. These fractures are located away from the first-formed TIR. Damage is generated at the inner surface of the LIR and propagates in the direction of thickness. Different from the NRZ, shear damage to the inner surface of the LIR promotes the accumulation of total damage;
(3) Fractures at the interface between the TIR and the NRZ are mostly due to shearing applied by the rib groove and radial tension during the formation of ribs. The damage variation at the outer surface and inner surface are the opposite at the start and end of the TIR groove filling stage, and the damage value at the outer surface of the TIR is the lowest;
(4) Given the huge demand for thin-walled complex components in the aerospace field, the development of precision spinning techniques will be the trend of the future. For this reason, the mechanism of damage formation and the prediction and control of fractures are very important research issues. Thus, we will optimize the spinning parameters and structure parameters of inner ribs to produce high-performance thin-walled tubular parts in subsequent works.

## Figures and Tables

**Table 1:** Some stress state characterization equations used in modified GTN models.

Authors	Stress State Characterization Equation
Nahshon and Hutchinson (2008) [18]	$g_{0}=1-\xi^{2}$
Xue (2008) [19]	$g_{\theta }=1-\frac{6\left|\theta \right|}{\pi }=1-\left|1-\frac{2}{\pi }\arccos\xi \right|$
Malcher et al. (2014) [20]	${g^{\prime }}_{0}(\xi ,\eta )={\left[1-\xi^{2}\right]}^{\frac{1}{\left|\eta \right|+k}}$
Jiang et al. (2016) [21]	$\omega_{\sigma }=\left\{\begin{array}{l} (1-\xi^{2})(1-c_{1})+c_{1}\\ (1-\xi^{2})(1+\frac{c_{1}}{k_{1}}\eta )-\frac{c_{1}}{k_{1}}\eta \\ 1-\xi^{2} \end{array}\begin{array}{l} \;\eta <-k_{1}\\ \;-k_{1}\leq \eta \leq 0\\ \;\eta >0 \end{array}\right.$

Note: ξ denotes the normalized third deviatoric stress invariant, θ is the Lode angle, η is stress triaxiality and *k*, *c*_1_ and *k*_1_ are the undetermined constants.

**Table 2:** Mechanical property parameters of annealed 2219 aluminum alloy tubular billet.

Parameters	*E* (GPa)	*ν*	*K* (MPa)	*n*	*ε*_0_
Value	68	0.3	291.8	0.219	0.0001

**Table 3:** Damage parameters of annealed 2219 aluminum alloy tubular billet.

Parameters	$\varepsilon_{N}$	$s_{N}$	$f_{N}$	$f_{c}$	${\varepsilon^{\prime }}_{N}$	${s^{\prime }}_{N}$	$D_{N}$	$D_{c}$	*K*_g_
Value	0.1	0.2	0.04	0.04	0.1	0.1	0.01	0.52	2.5

## Data Availability

Data are contained within the article.
